# Genotypes of glycoprotein B gene among the Indian symptomatic neonates with congenital CMV infection

**DOI:** 10.1186/s12887-019-1666-5

**Published:** 2019-08-22

**Authors:** Agniswar Sarkar, Dipanwita Das, Sabbir Ansari, Rajendra Prasad Chatterjee, Lopamudra Mishra, Biswanath Basu, Sanat Kumar Ghosh, Mala Bhattacharyay, Nilanjan Chakraborty

**Affiliations:** 1 Virus Unit [NICED-ICMR], GB4-1st Floor, ID and BG Hospital, 57, Dr. S. C. Banerjee Road, Beliaghata, Kolkata, West Bengal 700 010 India; 2 Dr. B. C. Roy Post Graduate Institute of Pediatric Sciences, 111, Narkeldanga Mail Road, Phool Bagan, Kankurgachi, Kolkata, West Bengal 700 054 India; 3 Department of Pediatric Nephrology, Nil Ratan Sircar Medical College and Hospital, 138, Acharya Jagadish Chandra Bose Road, Sealdah, Kolkata, West Bengal 700 014 India

**Keywords:** Cytomegalovirus, Molecular epidemiology, Genotyping, Polymerase chain reaction, Congenital infection, Glycoprotein B

## Abstract

**Background:**

Cytomegalovirus [CMV] is a causative agent of congenital infection worldwide and often leads to neurological deficits and hearing loss in newborns. Infants born with symptomatic congenital Cytomegalovirus infection [cCMV] are at significant high risk for developing adverse long-term outcomes. In this study, we look into the sequence variability of surface glycoprotein B [gB] encoding region in newborns with symptomatic CMV infection for the first time in Eastern region of India.

**Methods:**

576 suspected newborns from seropositive mothers were subjected to the study and ELISA was used to confirm CMV infection. Different genotypes and their subtypes were determined using multiplex nested-PCR. Viral load of different glycoprotein B [gB] genotypes was measured using RT-PCR. Sequencing and phylogenetic analysis was then performed using Bayesian interference.

**Results:**

The overall frequency of cCMV infection was 18.4%, where 16.0% neonates were symptomatic. Among the different gB genotypes, gB1 had the highest frequency [23.5%] and gB4 showed the lowest occurrence [5.8%]. 23.5% of symptomatic neonates had mixed genotypes of gB, probably indicating matrenal reinfection with CMV strains in Indian population. Significant genotypic clades [gB1-gB2-gB3-gB5] were grouped closely based on gene sequences, but the gB4 sequence was in the outlier region of the phylogenetic tree indicating the genetic polymorphism.

**Conclusion:**

This is the first study on cCMV genotyping and its phylogenetic analysis from Eastern Indian neonatal population. The study holds importance in the assessment of cCMV seroprevalence in global perspective. gB protein can be used as a potential therapeutic target against CMV infection.

## Background

Cytomegalovirus [CMV] has emerged as one of the leading cause of viral congenital infection and is a neglected problem worldwide including India. This is due to the fact that almost all of these congenital infections remain asymptomatic and may not be recognized at birth [1]. The immune status of the patients determines reactivation of latent CMV infection in several clinical cases. Reactivation of latent infection leads to an increased chance of recombination and development of novel CMV variants [2]. Congenital CMV infection is caused due to maternal transmission of new or reactivated latent infection to the fetus, at any gestational stage. Newborns may acquire CMV infection through congenital, intrapartum and antenatal routes of infection. Congenital CMV infection is transmitted transplacentally and may result in symptomatic or asymptomatic infection in neonates. Several epidemiological studies on the incidence of congenital Cytomegalovirus [cCMV] infections have been carried out in many countries of Europe and the United States of America [3]. Some limited number of seroepidemiological studies that has been conducted in the Indian population, show 80–90% prevalence of IgG antibodies in women of childbearing age [4, 5]. Infants with cCMV infection are categorized as symptomatic and asymptomatic based on presence and absence of clinical findings. Around 90% of children with cCMV infection do not show clinical abnormalities and are classified as asymptomatic, whereas only 10% of children showing clinical abnormalities are considered symptomatic [6–9].

Newborns with symptomatic infections are at severe risk for developing adverse neurodevelopmental sequelae [6]. Symptomatic cCMV infections are associated with intrauterine growth retardation [IUGR] [10], long-term neurological sequelae [i.e., cognitive and motor impairment, hearing loss, visual impairments], microcephaly [11], petechiae, jaundice, hepatosplenomegaly [11, 12], retinitis [4], cerebral abnormalities [13], thrombocytopenia, and fetal/infant death [13, 14]. Prevention and control of cCMV infection stands as an ongoing challenge [12, 15]. Traditional cCMV diagnosis is complicated, as serological evidence of active infection cannot be correlated with the clinical status [9]. The magnitude of the problem in India needs thorough and in-depth investigation. Serological assays for CMV-specific IgM have been previously used in several studies, for detection of congenital infection [1, 5, 16, 17]. Our study has been designed to address the problems of cCMV genetic variations among the neonates of the Eastern Indian region. CMV glycoprotein B [gB] [UL55] is a major envelope glycoprotein, which exists in the viral envelope and acts as proteolytically processed protein dimer on the membrane surface of all CMV-infected cells [18]. Apart from its role in virus entry and fusion, gB is also required for cell-to-cell proliferation. gB gene plays a key role in the “priming” of the transcriptional machinery in the host cell before viral replication begins [19]. Accordingly, gB gene might be a powerful cCMV pathogenicity marker for their potential implication in virulence and cell tropism. The present study is one of the first reports showing the prevalence of cCMV infection in symptomatic neonates of Eastern Indian population. We further examined the distribution of CMV-gB genotypes and it was attempted to associate the clinical and prognostic significance with the circulating genotypes. The study also holds a promising role in utilizing gB as novel marker of infection.

## Methods

### Study groups and clinical specimens

The study was carried out in the Virology Laboratory [Indian Council of Medical Research Virus Unit, Kolkata, West Bengal, India] between August 2014 and July 2016. In total, 576 live-born infants, born to CMV infected mothers, from different metropolitan hospitals and medical colleges were included in this study. The patients enrolled for the study included suspected newborns within 2 weeks of birth. Infants born to CMV infected mothers having present/active infection [tested positive for IgM] or past infection [tested positive for IgG] was the chosen population for the study. Majority of cases reported in hospitals were mixed population from both urban and rural areas, but mostly from low socio-economic strata.

### Sample collection, clinical assessment, screening and serodiagnosis

About 3–5 mL of blood specimens were collected from suspected newborns within 2 weeks after birth. Clinical data were collected from the information sent by the physicians. All serum samples were screened and the presence of CMV-IgM antibody was determined by enzyme-linked immunosorbent assay [ELISA] using the commercially available kit [Equipar SRL, Lombardia, Saronno, Italy] according to the manufacturer’s instructions. Neonates with positive IgM and CMV-DNA in blood within 2 weeks of life with clinical signs and symptoms were considered as symptomatic congenital CMV infection.

### Genomic DNA isolation and quantification

CMV DNA was extracted from 200 μl of plasma using the QIAamp DNA Blood MiniKit [QIAgen, Hilden, Germany] following manufacturer’s instructions. Extracted DNA from symptomatic newborns were subjected to PCR for the molecular genotyping through amplification and detection of gB genotypes.

### Estimation of viral load using real time PCR

Real-time PCR reactions and quantitative analysis were performed to measure CMV titre in specimens based on the earlier method described by Kubar et al., 2004 [20]. PCR amplifications were performed as individual assays for each sample. The sequences for the PCR primers and probes were designed using the PrimerQuest tool from Integrated DNA Technologies [IDT]. The TaqMan probes were labeled with 6-carboxyfluorescein [FAM] reporter dye at the 5′ end, and with the 6-carboxytetramethyl-rhodamine [TAMRA] quencher dye at the 3′ end as described previously. Amplification, data acquisition and all analyses were carried out using the ABI 7200 SDS [Applied Biosystems, Foster City, CA, United States].

### Statistical analysis

All live-born infants were systematically screened during the study period to determine the birth prevalence of symptomatic cCMV infection. The birth prevalence was defined as the number of infected infants divided by the total number of live-born infants. Fisher’s exact test was used to evaluate the statistical impact of different groups, and *p* values < 0.05 were considered significant at the 95% confidence interval. Statistical analysis was conducted using the Statistical Package for the Social Sciences [SPSS] 16.0 software [SPSS, Inc., Chicago, IL, USA].

### Primer designing for conventional CMV PCR

The CMV glycoprotein B [gB] gene sequence was extracted and retrieved from the laboratory strain AD169 [FJS27563] through the NCBI database. All open reading frames [ORF] were reanalyzed and recalculated by selecting the start codon through ORF finder [21, 22]. The most conserved domain was distinguished utilizing the NCBI-conserved domains database [NCBI-CDD] and BLASTp algorithms [23, 24]. CMV-specific internal primers for gB genotype was designed using Primer 3.0 [Table 1]. In the East Indian newborns, sequence variability of gB genotypes [gB1 to gB5] were amplified and analyzed using external primer and internal primers as described by Tarrago´ et al. [2].

**Table 1:** Sequences of oligonucleotide primers used for the amplification of CMV gB [UL55] encoded glycoprotein genes

Virus/genotypes Target genes	Primers and probes [5′ → 3′]
CMV-gB gene	External Primer:
	F: TTT GGA GAA AAC GCC GAC R: CGC GCG GCA ATC GGT TTG TTG TA
gB genotypes: gB1	Internal Primer:
	F: ATG ACC GCC ACT TTC TTA TC R: GTT GAT CCA CRC ACC AGG C
gB2	F: TTC CGA CTT TGG AAG ACC CAA CG R: GTT GAT CCA CRC ACC AGG C
gB3	F: TAG CTC CGG TGT GAA CTC C R: GTT GAT CCA CRC ACC AGG C
gB4	F: ACC ATT CGT TCC GAA GCC GAG GAG TCA R: GTT GAT CCA CRC ACC AGG C
gB5	F: TAC CCT ATC GCT GGA GAA C R: GTT GAT CCA CRC ACC AGG C

### Molecular diagnosis of gB genotypes by multiplex nested PCR [M-nPCR]

A standard strain of CMV [ATCC-AD169] was used as the positive control and amplification reactions were performed by the GeneAmp PCR system [Applied Biosystems, USA].

Genotypic distribution of gB gene was measured in 17 symptomatic cCMV infected neonates. For the first-round amplification of glycoprotein B [gB] gene, reaction mixtures consisted 12.5 μL of 2X ready master mixture [Takara, Japan], 5 μl of target DNA [60–65 ng], 10 pmol of each oligonucleotide primer, and molecular grade water [Sigma, USA] to a total volume of 25 μl. The conditions for amplification with external primers were 94 °C for 3 min, followed by 35 cycles of 94 °C for 45 s, 55 °C for 45 s, and 72 °C for 45 s, and the final extension at 72 °C for 5 min. For the second round PCR, 2 μL of each amplicon was added as template to the 12.5 μL of 2X ready master mixture [Takara, Japan], 10 pmol of each internal primer, and molecular grade water [Sigma, USA] to a total volume of 25 μl. The second round PCR program carried out at 94 °C for 3 min, followed by 35 cycles of 94 °C 45 s, 52 °C for 30 s and 72 °C for 45 s with a final extension at 72 °C 5 min. The amplified products were estimated by visual comparison with the standard DNA molecular markers and amplicons were generated after electrophoresis through 2.0% agarose gel, stained with 0.5 mg ml^-l^ ethidium bromide [Sigma Chemicals Co., USA] and visualized on Gel Documentation System [Biorad].

### DNA sequencing

Amplified gB gene products from each gB genotype was selected for sequencing from symptomatic newborns. The sequences obtained were visualized as computer traces using Chromas lite v1.62. All sequences were analyzed in both, forward and reverse directions.

### Bioinformatics and phylogenetic analysis

The nucleotide sequences of gB gene was checked, edited and analyzed by DnaBaser v3.0. Clustal omega with complete alignment mode was used for the multiple sequence alignment. Our sequences were compared with published sequences from Viper, DDBJ/EMBL/NCBI-GenBank database along with sequences of AD169, Merlin, Toledo, and Towne reference strains. Inter/intra-genetic distribution and their variations were analyzed and compared on local and global perspectives. Phylogenetic analysis was performed through Monte Carlo Markov Chains [MCMC] methods using Bayes’ theorem [25], which incorporated a model of evolution, quantified and addressed the source of uncertainty and was able to incorporate complex models of evolution. Bootstrap calculations were based on 1000 repetitions [26]. Binary matrix was calculated to reconfirm the intra-sequence distribution and polymorphism in different gB-genotypes in symptomatic neonatal samples. *Net* average distances between *groups* of taxa was calculated using the formula ***d***_**A**_ **=** ***d***_**XY**_ **– [*d***_**X**_ **+** ***d***_**Y**_**]/2**; where, *d*_XY_ is the average distance between groups X and Y, and *d*_X_ and *d*_Y_ are the mean within-group distances. The average of outgroups is shown with “**n/c**”. Analyses were conducted using the Poisson correction model [27].

## Results

**Fig. 1:**
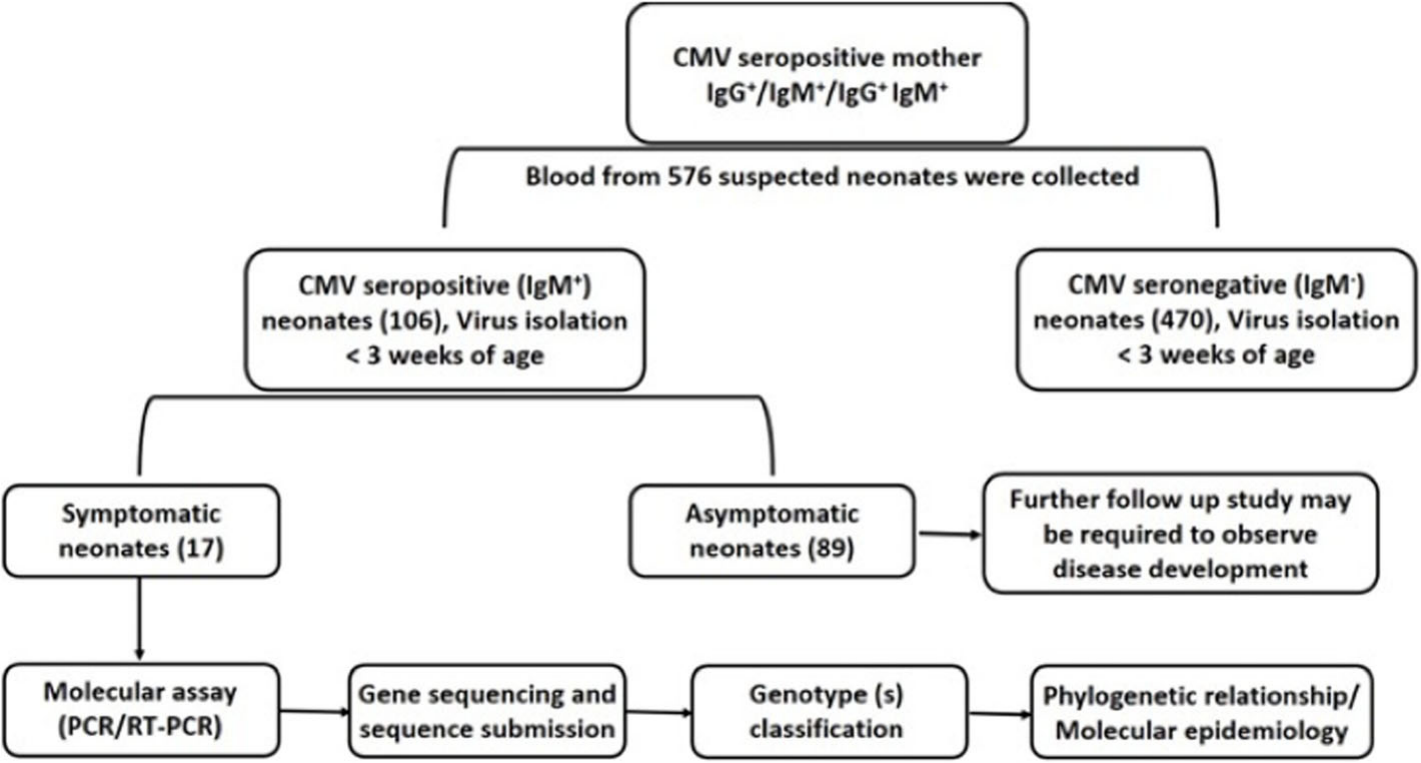
Proposed flow diagram showing the strategy for selection of patients study group for the maternal transmission of CMV and diagnostics for identification of congenitally infected neonates with suspected cytomegalovirus

Out of 576 newborns tested within 2 weeks after birth, the prevalence of cCMV infection was 18.4% [106/576] with or without symptoms. Among all infected newborns, 16.0% [17/106] of neonates were symptomatic and rest [*n* = 89] were asymptomatic [83.9%]. Symptomatic newborns with distinct clinical manifestations were enrolled in this study [Fig. 1]. Gender wise distribution among 17 symptomatic cCMV infections was 29.4% [5/17] in male and 70.5% [12/17] in female. A total of 89 asymptomatic cCMV positive neonates showed a gender-wise distribution of 35.9% males and 64% females. Thus, among a total of 106 CMV affected neonates, 69% were females and 37% were males. A dominance of CMV infection among females was thus established.

After confirming the CMV infection of newborns, studies on societal history of the families indicated that the cohort was restricted to a low-income group. It was observed that 52.9% [9/17] of mothers were working women. Though there was a higher prevalence of CMV-IgM among working women compared to housewives, the difference did not show significant association between occupation and IgM prevalence. Among clinical manifestations, hepatosplenomegaly was the most common feature [47.0%] followed by neonatal cholestasis/hyperbilirubinemia [41.1%], thrombocytopenia [35.2%], neonatal jaundice [29.4%], and IUGR [23.5%]. 11.7% of neonates were affected with bronchopneumonia [*n* = 3] and septicemia [n = 3] [Table 2]. Congenital cataract and hearing impairment was seen in 1 infant each. CT scan of the brain was performed in 3 infants as suggested by physicians and single infant showed bilateral sulcal calcification along with cholestatic jaundice and this particular finding was reported previously by our group [28].

**Table 2:** Distribution and frequency of clinical manifestations in symptomatic neonates with cCMV infection

Clinical manifestations	No. of NB affected [%]
Hepatosplenomegaly	8 [47.0]
Neonatal jaundice	5 [29.4]
Neonatal cholestasis	7 [41.1]
Bronchopneumonia	3 [17.6]
Septicemia	2 [11.7]
Thrombocytopenia	6 [35.2]
Intrauterine growth restriction [IUGR]	4 [23.5]
Microcephaly	3 [17.6]
Congenital cataract	1 [5.8]
Hearing impairment	1 [5.8]
Cerebral calcification	1 [5.8]

**Fig. 2:**
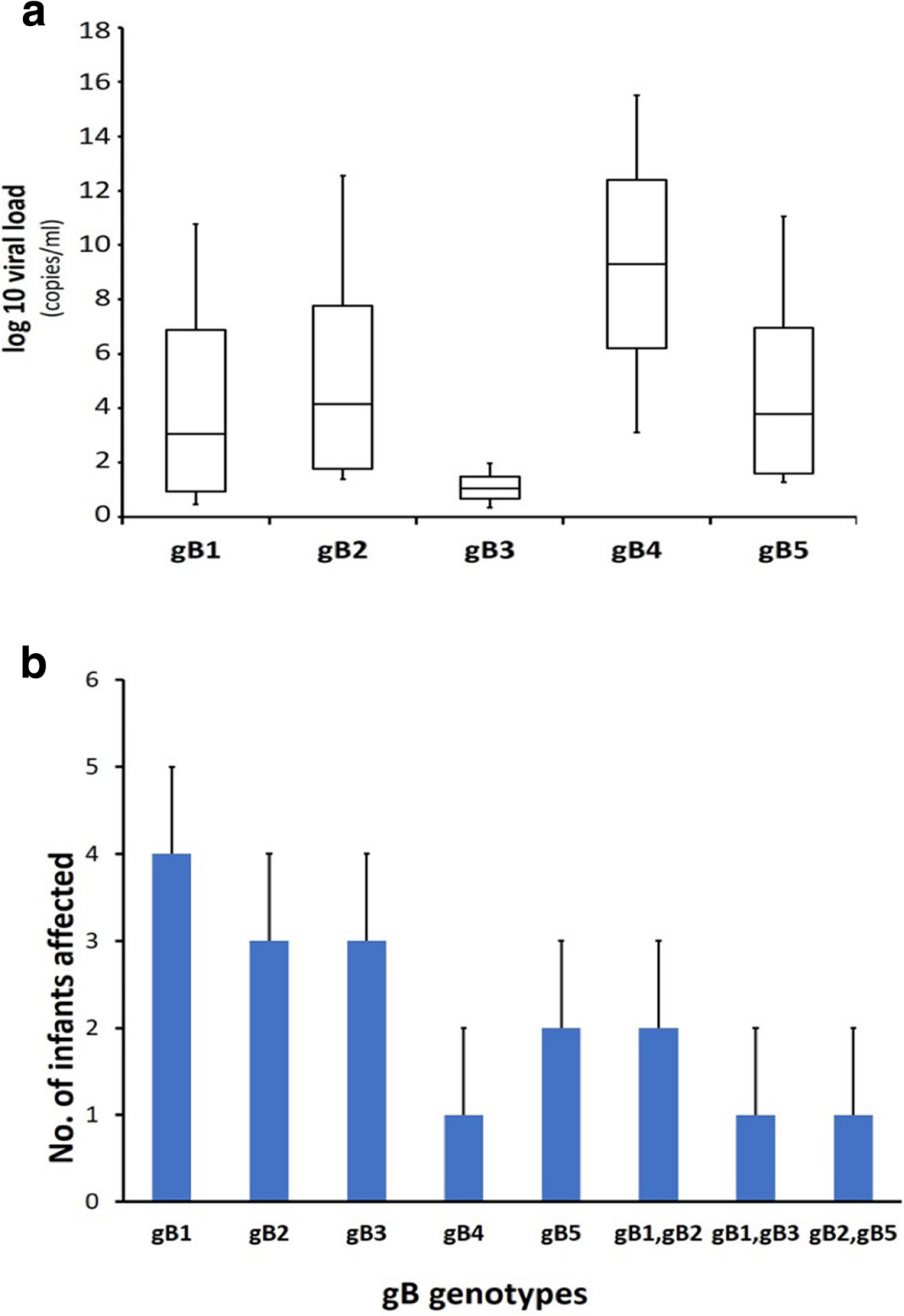
[**a**] Distribution of CMV-DNA load in congenitally infected symptomatic neonates. CMV DNA load of different gB genotypes and their subtypes was quantify using real time-PCR. Estimation of gB genotypes were corelated in five different subtypes. [**b**] Schematic representation for the genotypic distribution of CMV-gB pure and mixed subtypes in symptomatic newborns infected with CMV

CMV load was determined in 17 gB genotyped symptomatic cCMV infected samples [Table 3]. The overall median load of gB was 3.9 × 10^4^ log_10_/ml. Similarly, gB genotyped viral load was 2.1 × 10^3^ log_10_/ml for gB1, 2.4 × 10^4^ log_10_/ml for gB2, 3.8 × 10^3^ log_10_/ml for gB3, 3.1 × 10^4^ log_10_/ml for gB4 and 2.2 × 10^3^ log_10_/ml for gB5. Viral load of mixed genotypes were 3.9 × 10^3^ log_10_/ml, 3.9 × 10^4^ log_10_/ml, 4.8 × 10^4^ log_10_/ml for gB1, gB2; gB1, gB3; and gB2, gB5 respectively [Fig. 2a]. Genotypic prevalence of gB protein showed gB1 had highest frequency, followed by gB2 and gB3 while gB4 showed negligible occurrence [Fig. 2b]. Genotyping based on the gB region was determined by PCR from CMV-positive cultures where gB external primers was 100% positive in all cases [17/17]. In addition, the M-nPCR assay for gB genotyping was able to detect all samples containing single genotypes and as well as a mixture of genotypes. Table 3 summarizes the frequency of gB genotypes according to specimens along with clinical manifestations. The single genotype was distributed in 13 [76.47%] samples. gB1 was the most frequently occurring genotype and observed in 4 specimens [23.52%]. While 17.64% of gB2 and gB3 genotype was observed [*n* = 3] in each genotype, a single sample corresponded to gB4 [5.8%] and two for gB5 [11.76%]. However, a mixed-genotype infection was also detected in 4 [23.52%] cases. Among these mixed genotype infections, all were dual gB genotype infections and included 11.76% [2/17] gB1 and gB2, 5.88% [1/17] of gB1-gB3 and gB2-gB5 genotypes.

**Table 3:** Distribution and frequency of CMV-gB genotypes along with genotypic Clinical manifestations and viral copy numbers [log_10_] in congenitally infected symptomatic neonates

Sample No.	CMV load [log_10_/ml]	gB genotypes	Clinical significance
1.	3.2 × 10^3^	gB1, gB2	Hepatosplenomegaly, neonatal cholestasis, microcephaly thrombocytopenia, intrauterine growth restriction,
2.	2.7 × 10^2^	gB1	Hepatosplenomegaly, neonatal jaundice, neonatal cholestasis
3.	1.8 × 10^4^	gB2	Thrombocytopenia, intrauterine growth restriction, microcephaly
4.	3.6 × 10^3^	gB1	Hepatosplenomegaly, neonatal jaundice, neonatal cholestasis
5.	5.2 × 10^3^	gB1, gB2	Hepatosplenomegaly, neonatal cholestasis, microcephaly thrombocytopenia, intrauterine growth restriction,
6.	6.8 × 10^6^	gB2	Thrombocytopenia, intrauterine growth restriction, microcephaly
7.	3.8 × 10^3^	gB1, gB3	Hepatosplenomegaly, neonatal jaundice, neonatal cholestasis, bronchopneumonia, septicemia, thrombocytopenia
8.	2.8 × 10^4^	gB5	Septicemia, bronchopneumonia, thrombocytopenia
9.	4.5 × 10^2^	gB3	Bronchopneumonia, septicemia, thrombocytopenia
10.	3.8 × 10^3^	gB5	Septicemia, bronchopneumonia, thrombocytopenia
11.	1.6 × 10^5^	gB3	Bronchopneumonia, septicemia, thrombocytopenia
12.	6.1 × 10^3^	gB1	Hepatosplenomegaly, neonatal jaundice, neonatal cholestasis
13.	5.8 × 10^3^	gB2, gB5	Thrombocytopenia, microcephaly, septicemia, bronchopneumonia, hepatosplenomegaly, intrauterine growth restriction,
14.	3.4 × 10^3^	gB4	Congenital cataract, hearing impairment, microcephaly, hepatosplenomegaly
15.	2.4 × 10^5^	gB2	Thrombocytopenia, intrauterine growth restriction, microcephaly
16.	1.8 × 10^4^	gB3	Bronchopneumonia, septicemia, thrombocytopenia
17.	2.6 × 10^3^	gB1	Hepatosplenomegaly, neonatal jaundice, neonatal cholestasis

**Fig. 3:**
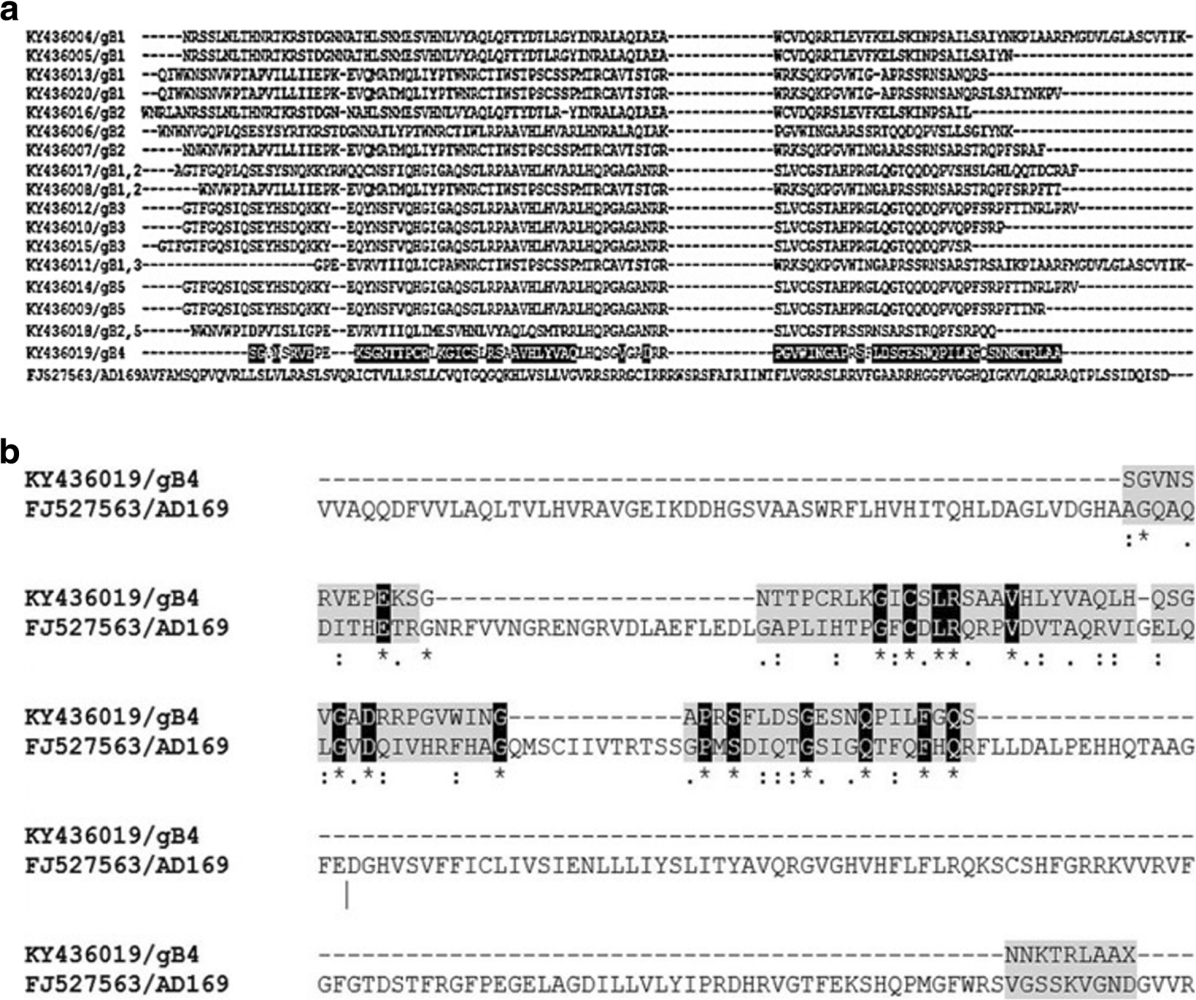
Amino acid sequence alignment of distinct sequences identified in the study. [**a**] Sequence alignment of gB4 genotype with the prototype strain, AD169. [**b**] Universal alignment of all the glycoprotein [gB] genotypes [gB1–5] with prototype strain, AD169

Submitted sequences of gB gene were compared to determine inter/intra genomic variations. GenBank accession numbers of the representative submitted sequences for 17 gB gene region from this study are KY436004 to KY436020. Sequences from the variable region of gB gene was compared to the published sequences of all gB genotypes. BLASTn analysis of our nucleotide sequences revealed about 98–100% similarity to prototype strains. Corresponding peptide sequences were translated using EMBOSS-Transeq tool [http://www.ebi.ac.uk/Tools/st/emboss_transeq/] and analyzed with the reference strains and presented in Fig. 3a and b.

**Fig. 4:**
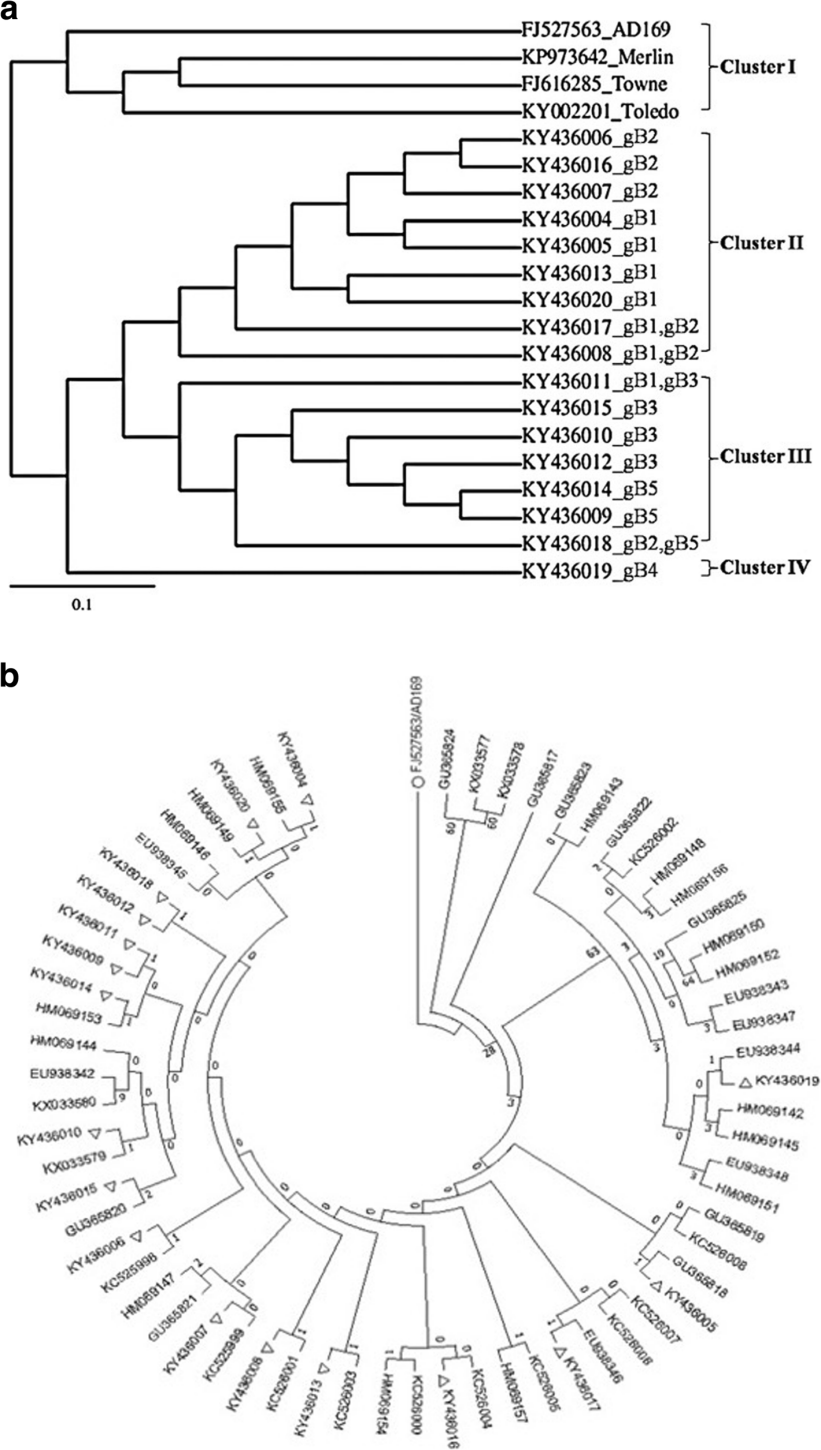
Phylogenetic tree based on the Neighbour-joining [N-J] method for the analysis of CMV gB gene sequences. [**a**] The intra genetic distribution along with reference strains; and the phylogenetic position of CMV-gB sequences isolated from eastern India and [**b**] Evolutionary relationships of taxa, where the optimal tree with the sum of branch length = 3.59972651 is shown. The percentage of replicate trees in which the associated taxa clustered together in the bootstrap test [1000 replicates] are shown next to the branches. The evolutionary distances were computed using the Maximum Composite Likelihood method and are in the units of the number of base substitutions per site. The analysis involved 65 nucleotide sequences. Codon positions included were 1st + 2nd + 3rd + Noncoding. All positions containing gaps and missing data were eliminated. [Ο represents CMV-AD169 reference strain and Δ represents isolates belonging to cCMV patients]

Similarity index was calculated through multiple sequence alignment [MSA]. Finally, the gB gene clusters and comparative sequences were analyzed based on the theory of Bayesian Probabilistic Approach 23, 24]. A total of 10,000 generations was taken for phylogenetic tree, which provided the bootstrap values in each branch after the analysis using Bayesian posterior probabilities, and was proportional to the number of substitutions per site. Different gene clusters were found with considerable distance, and each cluster was grouped within the same genotype except gB4 [Fig. 4a and b]. The evolutionary history was obtained by applying Neighbour-Join and BioNJ algorithms to a matrix of pairwise distances. The tree was drawn to scale, with branch lengths measured in the number of substitutions per site with all sequences. All nucleotide sequences were translated and compared within intra genotypic variations. Along with previously published sequences, five genotypic clades were identified in phylogenetic analysis. The phylogenetic tree indicated that genotype gB4 was distant, while genotypes gB1 with gB2 and gB3 with gB5 were more closely related [Fig. 4a]. Sequence comparison of amino acids in different types of symptoms-associated genotypes and predicted sequence alignment revealed that each of genotype was mostly homologous to each other [Fig. 4b]. It was observed that gB4 sequence had maximal significant variations among all the gB genotypes. The genotypic distribution was significantly variable from different geographical regions [29]. In Asia, Japanese genotypic distribution was found similar to the European genotypes [30, 31], while Chinese population showed a different pattern [32].

We further tried to compare the genotypic distribution of gB with the different clinical manifestations observed in the patients. Hepatosplenomegaly, neonatal cholestasis and thrombocytopenia was observed in 4 (23.52%) symptomatic patients with genotype gB1. Genotype gB2 was associated with IUGR and microcephaly in 3 (17.64%) infected infants. gB3 and gB5 was found in 3 (17.64%) and 2 (11.76%) patients respectively, who were suffering from septicemia and bronchopneumonia. Genotype gB4 was observed in 1 (5.8%) patient and was possibly related to various neurological disorders [e.g., Congenital cataract, hearing impairment] along with microcephaly and hepatosplenomegaly [Table 3]. This is a preliminary study conducted in a small cohort and needs to be conducted in a larger population to confirm the association of gB genotypes with specific clinical manifestations from Eastern India. Calculated binary matrix for each group was computed for all valid pairwise comparisons and results are displayed in the Table 4, where most polymorphic genotype [gB4- KY436019] belonged to the outgroup within cluster IV and matrix showing incalculable within group and shown as “n/c”.

**Table 4:** Estimates of Evolutionary Divergence between Sequences

FJ527563_AD169																	
KY436004	1.9																
KY436005	1.9	0.0															
KY436006	1.3	3.0	3.0														
KY436007	1.1	3.0	3.0	0.7													
KY436008	1.1	3.0	3.0	0.7	0.0												
KY436009	1.3	3.7	3.7	1.0	1.8	1.8											
KY436010	1.3	3.7	3.7	1.0	1.8	1.8	0.0										
KY436011	1.1	2.6	2.6	0.8	0.1	0.1	1.9	1.9									
KY436012	1.3	3.7	3.7	1.0	1.8	1.8	0.0	0.0	1.9								
KY436013	1.1	3.0	3.0	0.7	0.0	0.0	1.8	1.8	0.1	1.8							
KY436014	1.3	3.7	3.7	1.0	1.8	1.8	0.0	0.0	1.9	0.0	1.8						
KY436015	1.3	3.7	3.7	1.0	1.8	1.8	0.0	0.0	1.9	0.0	1.8	0.0					
KY436016	1.9	0.0	0.0	3.0	2.6	2.6	3.7	3.7	3.0	3.7	2.6	3.7	3.7				
KY436017	1.3	3.7	3.7	1.0	1.8	1.8	0.0	0.0	1.8	0.0	1.8	0.0	0.0	3.7			
KY436018	1.3	1.1	1.1	1.7	1.1	1.0	1.3	1.3	0.8	1.3	1.1	1.3	1.3	1.2	1.2		
KY436019	1.7	3.7	3.7	1.0	1.3	1.3	1.0	1.0	1.2	1.0	1.3	1.0	1.0	***n/c***	1.0	1.1	
KY436020	1.1	3.0	3.0	0.7	0.0	0.0	1.8	1.8	0.1	1.8	0.0	1.8	1.8	2.6	1.8	1.1	1.3

The number of amino acid substitutions per site from between sequences are shown. The analysis involved 18 amino acid sequences. All positions containing gaps and missing data were eliminated. There were a total of 44 positions in the final dataset. Evolutionary analyses were conducted in MEGA. The presence of n/c in the results denotes cases in which it was not possible to estimate evolutionary distances

## Discussion

cCMV is a leading cause of public health problem throughout the world including India. Asymptomatic infants affected with CMV infection have no apparent clinical symptoms and 10–15% of these children develop long-term sequelae [33–35]. The prevalence of congenital CMV infection may vary on the basis of the differences in a geographical region, depending on the racial, ethnic, socioeconomic background and diagnostic methods used to detect infection [36]. Due to a high seroprevalence of CMV in developing countries, diagnosis of cCMV infection in symptomatic patients is recommended to reduce infant morbidity, mortality and sequels [37]. Very limited information is available about the incidence and the history of this infection in India [4, 38]. There are no previous reports on molecular data for symptomatic cCMV infection from Eastern Indian region. This is the first genotyping study of cCMV-gB transmission among symptomatic neonates in Eastern India region, to the best of our knowledge. Numerous studies reported around 10–15% of neonates show relevant clinical signs with congenital infection during birth [39]. Our study showed that congenital CMV infection had a high prevalence in Eastern India, which was in corroboration with other parts of India and abroad. Since the symptomatic newborns are at a much higher risk for developing clinical complications, we chose to study the genotypic variation in this population. The asymptomatic newborns are at a lower risk for adverse neurodevelopmental sequelae and thus they may be considered for a follow-up study, to ensure a better management of asymptomatic disease burden. The present study shows that the prevalence of symptomatic cCMV infection was 16.03% from total CMV positive newborns. Kenneson and Cannon reported the overall birth prevalence of congenital CMV infection was 11.0% and varied considerably among different study populations [40]. Several studies also reported that about 13.5% of CMV infected newborns are born with symptoms [33, 41]. Another study has reported that 19.4% of babies were congenitally infected through CMV infection with various birth defects in India [29]. In this study, gender wise prevalence was measured in symptomatic as well as asymptomatic newborns. 29.4% males and 70.5% females were infected with CMV among symptomatic newborns. On the other hand, 35.9% males and 64.0% females were found asymptomatic. In both cases [symptomatic and asymptomatic], distribution frequency was higher in female patients as compared to male. Most frequently observed clinical symptoms were hepatosplenomegaly [47%] and neonatal cholestasis [41.1%], which was mostly associated with the gB1 genotype. These observations are in concordance with other previous reports [39, 40] [42, 43]. Shukla et al., [2015] found a significant pre-dominance of females (59.72%) among CMV positive patients [44]. Similar results were stated by Colugnati et al., [2007], where CMV IgG titre was higher in females [45]. A report by Firth et al., 2016 and Lachmann et al., 2018 also explained the higher incidence of CMV in female patients [46, 47]. Genetic polymorphisms of envelope glycoproteins among circulating CMV strains are generally considered as probable virulence indicators [48], and may be accountable for differential CMV tropism to precise cell types and differential capacity to distribute and interfere with normal tissue growth [30, 39]. Previous reports show that four gB genotypes of CMV are observed in congenital cytomegaly [48, 49]. Several studies reported that cCMV infections among Costa Rican, Indian and Chinese infants were caused by gB1, gB2, and gB3 genotypes [29, 42]. In a Polish study, cCMV infection occurrence of gB1, gB2, gB3, and gB4 genotypes was found to be about 43.7, 31.25, 25.00, and 12.5%, respectively [50]. A previous study showed high prevalence of gB1 and gB2 genotypes [50.0%], reduced prevalence of gB3 genotype [8.3%] and negligible incidence of gB4 genotype in affected subjects [49]. Our studies revealed the highest incidence of gB1 [23.5%], moderate incidence of gB2 and gB3 [17.6%], partial incidence of gB5 [11.7%] and negligible occurrence of gB4 [5.8%] among symptomatic neonates of eastern India. Interestingly, this was in concordance with genotypic distribution of cCMV infection from different geographical regions including Costa Rica, rest of India, China and South Hungary and also had gB1 as the most prevalent circulating genotype in these regions [4, 39, 42, 51]. Differential distribution of envelope glycoprotein genotypes circulating in discrete geographical regions among congenitally infected patients have been reported previously [2, 3]. Interestingly our studies revealed that in eastern India, gB1 genotype for monoinfection and gB1-gB2 for mixed infection is maximally prevalent in cCMV infection. Similar reports were observed in other studies [3]. Our study reports genotypic distribution of cCMV infection in Eastern Indian region for the first time. We report that 23.5% of symptomatic neonates had mixed genotypes of gB. This is an interesting finding and previous reports show occurrence of mixed genotypes of gB in geographical regions other than India, which we have discussed vividly. A probable reason for this finding suggests that maternal reinfection with CMV strains could be frequent in Indian populations. It has been observed previously, that presence of multiple gB genotypes could be a critical factor associated with severe clinical manifestations compared to the presence of a single gB genotype, in immunocompromised patients [52].

Further, it was observed that the symptomatic neonates with mixed genotype infection had detectable appreciable viral load. Previous studies have shown that infections with mixed genotypes increases the chances of progression to CMV diseases [52, 53].

Among the different circulating glycoprotein genotypes in symptomatic newborns of eastern India, gB1 was maximally prevalent followed by gB2 and gB3, while gB4 showed negligible occurrence. Similar studies reported that gB1 was the more frequent genotype in infants infected congenitally in Hungary [39], Italy [51, 54] Japan] [30, 31], and the United States [50]. gB2 was found to be a major genotype transmitted congenitally in Australia [55]. Picone et al. also found a similar distribution of genotypes gB1, gB2, and gB3 in CMV strains recovered from amniotic fluid samples in France [56].

Intra-host viral diversity and genomic variation have recently been characterized by next-generation sequencing, showing similar genomic variability in different RNA viruses [48, 49] [57, 58]. Mixed populations of the glycoprotein genotype have been previously documented in congenitally infected neonates and in pregnant women [59]. Our study describes the primary circulating genotypes in the affected neonates, and these genotypes probably play vital roles in disease severity in this geographical region.

As the genotypic distribution of CMV varies among different geographical regions [60], we compared the symptomatic cCMV-gB genotypes with those reported previously. Present study was formulated to address the polymorphisms of CMV genes encoding different gB genotypes in symptomatic cCMV infections. Results from this study was further analyzed and confirmed through DNA sequencing and phylogenetic analysis using Bayesian interference. According to the algorithm for sequence comparison, gB variable region was compared to the other available repository sequences in the database. Phylogenetic analysis from our study suggested that gB1 and gB2 may have common evolutionary origin in Eastern Indian region as both are clustered together in the same clade. However, gB3 and gB5 gene cluster was found in the different clade with considerable distance. The four clusters [gB1, gB2, gB3 and gB5] were grouped within the same genotype except gB4. In our study, we observed that gB4 positive neonates were related to specific neurological disorders like congenital cataract and hearing impairment. These particular findings are different from the study which was conducted in the other part of India, where they have found that same symptoms were related to gB2 genotype [27]. Comparison of the sequences on global perspective portrayed a distinct pattern of gB genotypes, where eastern Indian sequence was clustered within the same genotype along with other gB genes of various province. Interestingly, this revealed that CMV gB gene is composed of highly conserved regions.

This is the first report on genotypic variations in cCMV infection from eastern India region. gB4 genotypic polymorphisms have been associated with variable clinical outcomes. Our study focuses on the initial correlation of the genotype variability with the different diseases in symptomatic neonates from eastern India. CMV is able to establish latency and evade immune surveillance. This presents particular challenges in the development of effective vaccination as CMV genome displays great genetic heterogeneity. The purified gB protein stands as an important candidate for vaccine development against CMV infection. Neutralizing antibodies against CMV infection was produced when the purified protein was injected in combination with MF-59 oil-in-water adjuvant in humans for a fixed regimen of six months [34]. Thus, there is a constant need for upgrading the information on molecular epidemiology of CMV in different population and geographical regions, which would help in developing effective universal vaccines for the prophylactic treatment of CMV in humans.

## Conclusion

We believe to the best of our knowledge that this is the first molecular evaluation on cCMV-gB genotyping and their phylogenetic position in east Indian symptomatic neonatal population. This is important for assesement of the cCMV seroprevalence in the global perspective. This study also highlighted the efficacy of PCR-based techniques in following possible transmission of CMV within communities. However, it is worth mentioning that we had limited sample size for the seroprevalence screening. Taken together, the present study is extremely useful in developing gB protein as a prospective therapeutic target for CMV. We strategize to carry out a follow up study for the asymptomatic cCMV infected newborns to have a bird’s eye view of the disease in the global perspective. Similar disease specific genes can be identified for developing curative approaches for other viral infections.

## Data Availability

Complete raw data is available on request. Kindly contact the corresponding authors for raw data.

## Abbreviations

ATCC-AD169: American Type Culture Collection-AD169 cytomegalovirus strain
BLASTn: Basic Local Alignment Search Tool for nucleotide databases
cCMV: Congenital Cytomegalovirus
CDD: Conserved domains database
CMV: Cytomegalovirus
DDBJ: DNA Data Bank of Japan
EMBL: European Bioinformatics Institute
gB: Glycoprotein B
IUGR: Intrauterine growth retardation
MCMC: Markov Chains
NCBI: National Center for Biotechnology Information
RT-PCR: Real time PCR
UL55: unique long gene 55
